# Building biointegration of Fe_2_O_3_–FeOOH coated titanium implant by regulating NIR irradiation in an infected model

**DOI:** 10.1016/j.bioactmat.2021.06.029

**Published:** 2021-06-29

**Authors:** Yang Xue, Jun Chen, Tiexin Ding, Mengting Mao, Shengbo Zhu, Jianhong Zhou, Lan Zhang, Yong Han

**Affiliations:** a State-key Laboratory for Mechanical Behavior of Materials, Xi'an Jiaotong University, Xi'an 710049, China; b Department of Osteology, Xi'an People's Hospital (Xi'an No. 4 Hospital), Xi'an, 710100, China; c Department of Osteology, Tangdu Hospital, Fourth Military Medical University, Xi'an, 710038, China; d Institute of Physics & Optoelectronics Technology, Advanced Titanium Alloys and Functional Coatings Cooperative Innovation Center, Baoji University of Arts and Sciences, Baoji, 721016, China

**Keywords:** Photothermal, Fe_2_O_3_–FeOOH, Antibacterial, Biointegration, Soft tissue

## Abstract

Killing bacteria, eliminating biofilm and building soft tissue integration are very important for percutaneous implants which service in a complicated environment. In order to endow Ti implants with above abilities, multifunctional coatings consisted of Fe_2_O_3_–FeOOH nanograins as an outer layer and Zn doped microporous TiO_2_ as an inner layer were fabricated by micro-arc oxidation, hydrothermal treatment and annealing treatment. The microstructures, physicochemical properties and photothermal response of the coatings were observed; their antibacterial efficiencies and cell response *in vitro* as well as biofilm elimination and soft tissue integration *in vivo* were evaluated. The results show that with the increased annealing temperature, coating morphologies didn't change obviously, but lattices of β-FeOOH gradually disorganized into amorphous state and rearranged to form Fe_2_O_3._ The coating annealed at 450 °C (MA450) had nanocrystallized Fe_2_O_3_ and β-FeOOH. With a proper NIR irradiation strategy, MA450 killed adhered bacteria efficiently and increased fibroblast behaviors via up-regulating fibrogenic-related genes *in vitro*; in an infected model, MA450 eliminated biofilm, reduced inflammatory response and improved biointegration with soft tissue. The good performance of MA450 was due to a synergic effect of photothermal response and released ions (Zn^2+^ and Fe^3+^).

## Highlights

• Nanocrystallized Fe_2_O_3_–FeOOH layer endows Ti with good photothermal response.
• With NIR irradiation, Fe_2_O_3_–FeOOH layer improves biointegration in an infected model.
• Photothermal response combined with released ions gives implants good performance.

## Graphical abstract

**Figure:**
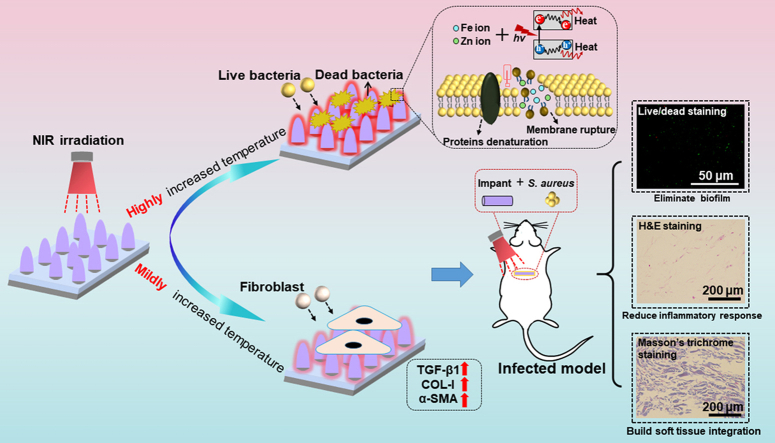

## 1 Introduction

Ti and its alloys with excellent mechanical properties, corrosion resistance and biocompatibility have been widely applied in intraosseous transcutaneous implants, such as dental implants and percutaneous prosthetics. Besides good osseointegration with host bone, biointegration with soft tissue is prerequisite for percutaneous implants to prevent bacterial infection and epithelium downgrowth [1,2]. However, Ti is bioinert and lacks antibacterial activity. It can't improve tissue-forming cell response, and bacteria may adhere on implant surfaces prior to cells, form biofilm and impede biointegration. After postoperative anti-infection management (e.g. intravenous antibiotics), most of bacteria can be killed and weak biointegration may form on percutaneous parts in initial period. However, due to the relatively open and complicated service environment, Ti implants are at high risk of infection and the biointegration can be destroyed easily. So, an ideal coating, which can timely kill the invasion bacteria and help to rebuild soft tissue integration after bacteria eradication during the long service life is needed.

Antibacterial agents (e.g. antibiotics, polypeptide and metal ions) loaded on implant surfaces can kill bacteria and reduce infection efficiently [[3], [4], [5]]. However, they generally have dose-dependent antibacterial activity. A locally high concentration of antibacterial agent is beneficial for prolonging antibacterial activity, but also induces cytotoxicity [6]. Light-based therapy, with its controllability, noninvasiveness and efficiency, offers therapeutic benefits that can improve patient compliance. Recently, photothermal therapy (PTT) by near-infrared (NIR) light irradiation has been used in tumor diagnosis and therapy in clinical and studied in the application of antibiosis [[7], [8], [9], [10]]. Under light irradiation, temperatures of photothermal agents increase due to the nonradiative relaxation of excited electrons. A locally high temperature (>50 °C) can induce bacterial protein denaturation and irreversible membrane destruction, resulting in bacteria death [10]. With the increased temperature and prolonged action time, sterilization of bacteria is better [8,11].

Extra heating treatment at a mild temperature was also reported to enhance bone formation-related cell response. For example, proliferation and differentiation of MC3T3-E1 cells were accelerated after 1 min treatment at 42 °C for 3 days [12]; hBMSC up-regulated BMP expression and down-regulated Noggin by activating phosphorylation of Smad 1 and Smad 5 after 1 min treatment at 42 °C for 3 days [12,13]; vimentin expression of MSCs was improved and MSCs preferentially differentiated into fibroblasts by 3 min treatment at 42 °C for 3 days [14]. When implants penetrate through soft tissue, fibroblasts around the wounds are activated and move to implant surfaces. They proliferate and secrete collagen I (Col-I) to remodel collage matrix, forming new soft tissue [1,15]. Whether fibroblast behavior and soft tissue response could be improved at a mildly increased temperature haven't been explored yet.

**Fig. 1:**
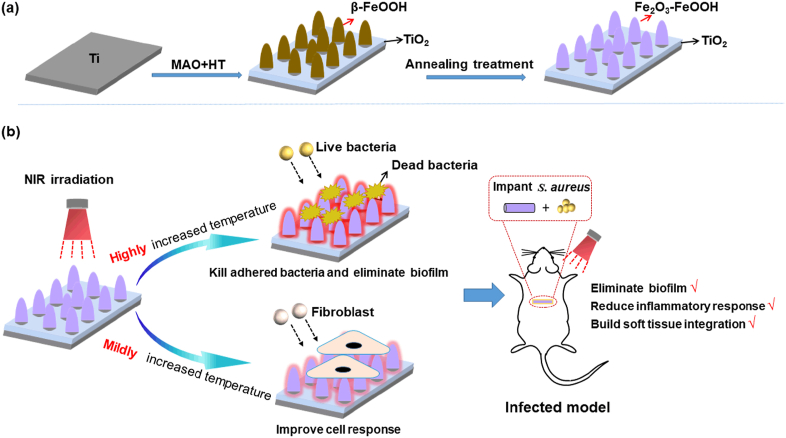
(a) Schematic illustration of the coating fabrication, (b) the coating was expected to kill *S. aureu*, eliminate biofilm, improve fibroblast response and accelerate soft tissue integration in an infected model by regulating NIR irradiation.

Photothermal agents usually applied in PTT are noble metal nanoparticles or nano-architectures (e.g. Ag nanospheres [16], Au nanorods [17,18] and Au based nano-architectures [19]), red and black phosphorus [[20], [21], [22], [23]], C-based nanocomposites (e.g. carbon dots [24,25], graphene oxide [12,26]) and sulfides (e.g. CuS [27,28], Bi_2_S_3_ [29], WS_2_ [30] and MoS_2_ [[31], [32], [33], [34]]), *etc*. However, noble metal nanoparticles or nano-architectures are clinically hindered by their biocompatibility and tissue persistence [19]; stability of red or black phosphorus needs to be settled [21,22]; preparation of coatings containing carbon dots [24,25] or graphene oxide is complicated [12,26]; metal sulfides have outstanding photothermal effect, but the cytocompatibility of released sulfur needs to be seriously considered [27,30,35]. In recent years, metallic oxides, especially iron oxides have been gradually studied due to their photo-related performance. They are biocompatibility and have good photothermal response, which have been widely used in magnetic resonance imaging and tumor ablation [[36], [37], [38]]. Unfortunately, iron oxides studied are usually powders, and only a few studies reported the preparation and photocatalysis activity of iron oxide coatings [[39], [40], [41]]. In our previous work, β-FeOOH layer was prepared and formed heterojunctions with TiO_2_ on Ti. Under light irradiation of 635 nm, reactive oxygen species formed by the heterojunctions inhibited 80% of *S. aureus* [42]. However, tissue penetration depth of 635 nm irradiation isn't deep enough (about 0.6 cm) and irradiation can't achieve implant surfaces when the covering soft tissue is relatively thick. It is known that NIR 808 nm irradiation with longer wavelength can penetrate soft tissue much deeper than 635 nm and is widely applied in PTT [13]. Fe_2_O_3_ has a good photothermal response and can be transformed from β-FeOOH by annealing treatment [43]. Based on the above studies, we initially prepared a β-FeOOH/TiO_2_ coating on Ti implant by a hybrid process of micro-arc oxidation (MAO) and hydrothermal treatment (HT), and then annealed coating samples at different temperature to make β-FeOOH transform to Fe_2_O_3._ The microstructures, released ions and photothermal properties of different samples were investigated; with 808 nm irradiation, their antibacterial activities against *S. aureus* and fibroblast response *in vitro* as well as inflammation response and soft tissue integration in an infected model were studied (Fig. 1). It is demonstrated that by controlling local temperature, the nanocrystallized Fe_2_O_3_–FeOOH coated Ti implants could kill *S. aureu*, eliminate biofilm, improve fibroblast response and accelerate soft tissue integration effectively*.*

## 2 Experimental methods

### 2.1 Preparation of the coatings

Commercial pure Ti plates (Ø 14 × 2 mm) and pillars (Ø 1.4 × 10 mm) were polished and ultrasonically cleaned in acetone, alcohol, and distilled water sequentially. They were micro-arc oxidized (MAOed) to form Zn doped TiO_2_, and then hydrothermally treated (HTed) with 1 M NaOH as detailed in our previous work [15,42]. The obtained samples were washed and subjected to HT again in 20 ml of an aqueous solution containing 0.5 M FeCl_3_·6H_2_O at 120 °C for 2 h. The obtained samples were named as MH. Finally, a part of MH samples were annealed in a muffle furnace for 2 h at 350 °C, 450 °C and 550 °C, respectively, and they were labeled as MA350, MA450 and MA550 according to the annealing temperature.

### 2.2 Structural characterization of the coatings

Surface morphologies and element compositions of the samples were characterized by field emission scanning electron microscopy (FESEM, SU6600, Hitachi, Japan) equipped with an energy dispersive X-ray spectrometer (EDX, DX-4, Philips, Netherlands). Phase compositions of coatings were analyzed using X-ray diffraction (X'Pert PRO, PANalytical Co. Netherlands) in θ-θ geometry (XRD) with Cu Kα rays. The chemical states were determined by X-ray photoelectron spectroscopy (XPS, Kratos Axis Ultra DLD, UK), and corrected using C 1s (hydrocarbon C/C, C/H) at a binding energy of 284.6 eV. The nanograins on different samples were initially scratched and dispersed in alcohol, and then dropped on a copper film and dried. Finally they were observed by a transmission electron microscope (TEM, JEM-2100F, JEOL, Japan) operating at 200 kV. The UV–vis–NIR absorption spectra from 300 to 800 nm for different samples were acquired on a UV–vis spectrophotometer (UV-3600, Shimadu, Japan) with fine BaSO_4_ as the reflectance standard.

### 2.3 Ion releasing of the coatings

Ions released from different coatings were measured by inductively coupled plasma-mass spectrometry (ICP-MS, PerkinElmer, NexION350D, USA). Five specimens were immersed in 5 mL 0.9 wt% NaCl aqueous solution at 37 °C for different days. At the pre-determined time points, the leaching liquids were collected and the solution was replaced. The concentrations of Fe and Zn ions released were measured. Three samples from each group were measured.

### 2.4 Photothermal response of the coatings

The photothermal response of different sample was characterized with an infrared thermal imaging system. Briefly, the sample in air or immersed in 0.5 mL of phosphate buffer saline (PBS) was irradiated under an 808 nm laser for 10 min and the temperatures were detected by a thermal camera. Three samples from each group were measured.

### 2.5 *In vitro* antibacterial test

Gram-positive *Staphylococcus aureu*s (*S. aureus*, ATCC25923) were cultured in beef extract/peptone (BEP, HopeBio, China) medium at 37 °C for 12 h. Then, they were adjusted to a concentration of 10^5^ CFU/mL in phosphate buffered saline solution (PBS) or nutrient broth. For the initial bacteriostasis, each sample was placed in 24-well plate, added with 1 mL of bacterial PBS suspension, and irradiated with 808 nm NIR laser at a power density of 0.5 W/cm^2^ for 7 min. After the treatment, the samples were ultrasonically treated at 40 W for 10 min, and viable bacteria were counted using a plate-counting method according to GB/T 4789.2 protocol. For elimination of biofilm, 1 mL of bacterial nutrient broth suspension was added on each sample and incubated at 37 °C for 24 h to form a biofilm on sample surface. Afterward, a part of samples were irradiated with 808 nm NIR laser for 7 min at a power density of 0.5 W/cm^2^. Then all samples were washed with PBS to remove non-attached bacteria and ultrasonically treated to isolate bacteria, and the viable bacteria were counted via a plate-counting method. The formula for calculating antibacterial ratio is: *C* = (*A*-*B*)/*A* × 100%; where *C* indicates antibacterial ratio; *A* and *B* are the numbers of viable bacteria on the Ti and experimental group. Live/Dead Baclight Bacterial Viability Kit (L13152, Invitrogen, France) was also used to evaluate the antibacterial efficacies of each coating with or without light irradiation. The specimen was rinsed three times with PBS, stained with SYTO9 and propidium iodide dyes for 20 min in darkness, and then biofilms were observed by an epifluorescence microscope (SMZ745 T, Nikon, Japan).

### 2.6 *In vitro* cytocompatibility evaluation

#### 2.6.1 Cell culture

Mouse fibroblast cell line (L-929) purchased from the Institute of Biochemistry and Cell Biology of Chinese Academy of Sciences (Shanghai, China), were inoculated in a humidified atmosphere incubator with 5% CO_2_ at 37 °C. The complete medium containing α-MEM (Hyclone, USA), 10% fetal bovine serum (Gemini, USA), 1 mM sodium pyruvate (Sigma, USA) and 15 mM NaHCO_3_ was refreshed every two days.

#### 2.6.2 Cell viability assay

500 μl of fibroblast suspension (1 × 10^4^ cells mL^−1^) was seeded on different samples and cultured with or without NIR irradiation for 1, 3 and 7 days. At the prescribed time point, viability of cells was assessed by Alamar Blue (Sigma, USA) assay according to the instructions and the absorbance was measured by a Multiscan Spectrum (Multiskan FC, Thermo, America). The cell statuses were also evaluated by Live/dead viability/cytotoxicity kit (Invitrogen, France) after 1 and 3 days of incubation according to the User Instruction, and epifiuorescence images were obtained by an Olympus BX52 microscope (SMZ745T, Nikon, Japan). The cell morphologies on different samples after incubation for 1 day were also evaluated by fluorescence staining of actin cytoskeleton, focal adhesion, and nuclei according to the operation manual as detailed elsewhere [44] and observed by an epifiuorescence (SMZ745T, Nikon, Japan).

#### 2.6.3 Quantitative real time-polymerase chain reaction (qRT-PCR)

Fibroblasts (1 × 10^4^ cells) were cultured on different samples with or without NIR irradiation after 7 days, respectively. After being rinsed with PBS for 3 times, total RNA was extracted from each sample using the Trizol reagent (Life Technologies, USA) and reversed transcribed into complementary DNA with PrimeScript RT reagent kit (Abcam, UK). The expression of key fibrogenic differentiation markers (α-SMA, TGF-β1, and Col-I) were evaluated by detection system (LightCycler 96, Roche, Switzerland) with SYBR1Premix ExTMTaqII (TaKaRa, Japan). Their sequences of specific primer sets were displayed in Table S1. The relative expression levels of the gene were determined using the 2^−ΔΔCT^ method, and the GAPDH expression was used to normalize the interested genes.

#### 2.6.4 Extracellular collagen secretion

Collagen secretion of fibroblasts cultured on each sample after 7 days with or without NIR irradiation was quantified by Sirius Red Staining according to the manual instruction. Three samples from each group were measured.

### 2.7 *In vivo* experiments

All animal experiments were conducted according to ISO 10993–2:1992 animal welfare requirements and were approved by the committee for Institutional Animal Care and Use of Xi'a Jiaotong University.

#### 2.7.1 Antibacterial activity and inflammatory response

Before surgery, Ti pillars (Ø1.4 mm × 1 cm) with or without coating were incubated in *S. aureus* suspension (1 × 10^8^ CFU/mL) for 6 h to form biofilm and dried in air for 10 min. Male SD rats about 200 g were randomly assigned to three groups and anesthetized with an intraperitoneal injection of 2.5 wt% pentobarbital sodium solution. After rats were shaved the dorsal hair and cleaned with 10% povidone-iodine, holes about 2 cm in length were drilled medially across the subcutaneous soft tissue using a Kirschner wire (Ø 2.0 mm). Each prepared pillar was inserted into a hole, and every rat had 8 samples, as illustrated in Figs. S1(a) and (b). After surgery, a part of samples were irradiated with the 808 nm laser (2.5 W/cm^2^) for 10 min everyday (Fig. S1(c)). After 1 and 4 days of implantation, rats were euthanized, pillars were taken out and rolled on agar plate and then placed at a temperature of 37 °C for one day to observe the growth of the bacterial colonies. Soft tissues around the implant were harvested for quantifying the expression of inflammatory factors (IL-1β, IL-6 and iNOS) by qRT-PCR according to section 2.6.3. The primers for the target genes were listed in Table S1. The skin tissues containing the implant were harvested after 4 days of implantation and fixed with 4% paraformaldehyde for histological examination by hematoxylin and eosin (H&E) staining. Digital microscopic image was captured on a microscopy (SMZ745T, Nikon, Japan).

#### 2.7.2 Tissue integration

The initially surgical procedure was the same as section 2.7.1. After implantation for 4 days, a part of coating samples were continued to irradiate by NIR at 1 W/cm^2^ for 3 min each day in the next four days. After 7 days of implantation, the rats were euthanized, the subcutaneous tissues were retrieved and α-SMA, TGF-β1 and COL-I were quantified. The primers for the target genes were listed in Table S1. The soft tissue with samples were examined by Masson's trichrome staining to observe the collagen deposition.

### 2.8 Statistical analysis

All data were presented as mean ± standard deviation and were analyzed via SPSS 14.0 software (SPSS, USA). In addition, the differences between the group means were evaluated via one-way ANOVA followed by a Student–Newman–Keuls post-hoc test. P < 0.05 and <0.01 were significant and highly significant differences, respectively.

## 3 Results and discussion

### 3.1 Microstructures and characterization of the coatings

**Fig. 2:**
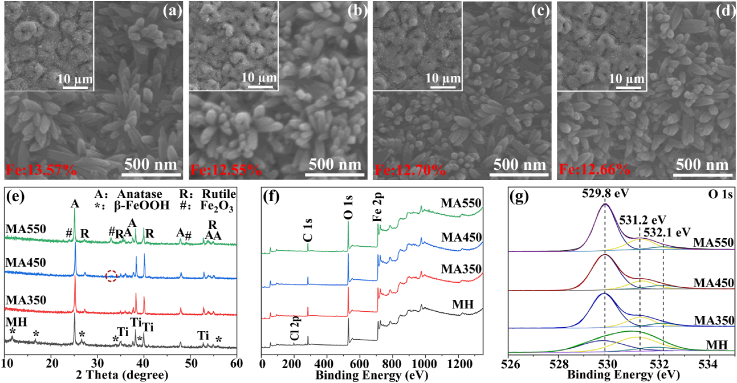
Surface morphologies of different samples: (a) MH, (b) MA350, (c) MA450 and (d) MA550, average Fe contents were inserted in the corresponding images; (e) XRD patterns of different samples; (f) Full XPS spectra and (g) high-resolution spectra of O 1s for different surfaces.

Fig. 2(a) shows the surface morphology of MH. β-FeOOH nanograins with an average diameter of 100 nm and length of 300 nm as an outer layer were homogeneously distributed on the porous MH surface. After annealing treatment, surface topographies in nano and micro scales as well as Fe and Zn contents have no obviously change (Fig. 2(b)–(d) and Table S2). The nanograin layers are all about 400 nm in thickness and Fig. S2 shows the cross-sectional SEM morphology of MA450 as an example. Phase compositions of different coatings were detected by XRD as shown in Fig. 2(e). MH contains anatase, rutile and β-FeOOH (JCPDS no. 34–1266) in accordant to our previous work [42]. After annealing treatment at 350 °C (MA350), the diffraction peaks of β-FeOOH disappear, and no new peak is observed. When annealing temperature increases to 450 °C (MA450), a peak of Fe_2_O_3_ (JCPDS no. 33–0664) appears (marked with a circle in Fig. 2(e)); with the temperature further increases (MA550), peaks of Fe_2_O_3_ grow sharply, indicating the transformation from β-FeOOH to Fe_2_O_3_ during the annealing treatment. XPS spectra are used to further reveal surface chemical compositions and elemental states on different surfaces. The full spectra (Fig. 2(f)) show that MH contains Fe, O, Cl and C. Fe, Cl and O are contributed by β-FeOOH and C comes from contaminant. After annealing treatment, the peaks of Cl decrease in strength for MA350 and MA450, and it even disappears for MA550. O 1s high-resolution spectra for different surfaces are shown in Fig. 2(g), and they were divided into three Gaussian-Lorentz component peaks. The peak at 529.8 eV is assigned to Fe–O; the peak at 531.2 eV corresponds to –OH in β-FeOOH; the peak at 532.1 eV attributes to the absorbed H_2_O (Fig. 2(g)). With the increased annealing temperature, the peak of Fe–O becomes stronger, whereas, the peak of –OH weakens. It further confirms the transformation from β-FeOOH to Fe_2_O_3_ during the annealing treatment. Specially, the ratio of Fe_2_O_3_/FeOOH on MA450 was estimated by the integral area changes of peaks at 529.8 and 531.2 eV (detailed in supporting information), and it is about 4.2.

As reported in our previous work [42], microporous TiO_2_ doped with Zn^2+^ is prepared on Ti by MAO; during HT in alkali solution, a layer of OH^−^-riched titanium oxide hydrate forms on TiO_2_ surface; OH^−^ ions react with Fe^3+^ ions to form β-FeOOH nucleis when TiO_2_ is subsequently HTed in Fe^3+^ containing solution; β-FeOOH nucleis grow with the prolonged HT or increased temperature. MH has a bigger average size of β-FeOOH nanograins and more Fe content compared to those in our previous work [42], which should be due to a higher HT temperature. β-FeOOH has a unique tunnel-type structure along the [010] direction, and when β-FeOOH formed in Cl^−^ containing solution, its tunnel sites are partly occupied by Cl ions [45]. During the annealing treatment, β-FeOOH gradually loses hydroxides and transforms to Fe_2_O_3_, resulting in a decreased amount of Cl (Fig. 2(f)).

**Fig. 3:**
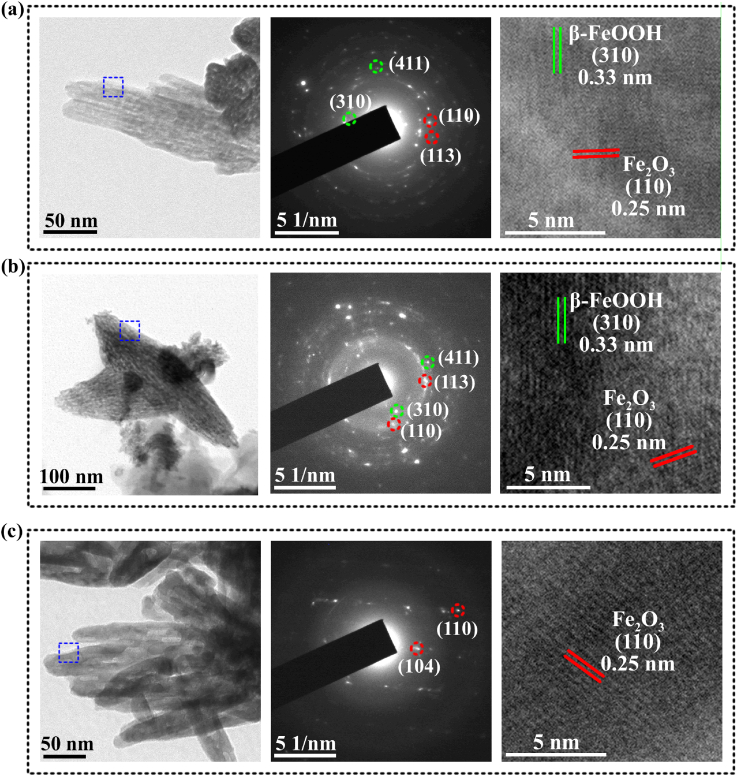
TEM micrographs of bright-field, corresponding SAED pattern and HRTEM images of the nanograins scratched from different samples: (a) MA350, (b) MA450 and (c) MA550.

Nanograins on MH surface are monocrystalline β-FeOOH, which were observed by TEM in our previous work [42]. The microstructures of nanograins on annealed surfaces were observed by TEM herein as shown in Fig. 3. Nanograins on different coatings are all rice-granule with similar size. For MA350 (Fig. 3(a)), the corresponding SAED pattern shows discrete circles and halation, indicating that grain lattices are randomly orientated and not well crystallized. The diffraction spots and rings are ascribed to β-FeOOH and Fe_2_O_3_ according to JCPDS card no. 34–1266 and 33–0664. For example, d-spacings of the rings marked with green circles have values of 0.333 nm and 0.195 nm, which are identified as (310) and (411) diffractions of β-FeOOH, respectively; the d-spacings of rings marked with red circles have values of 0.251 nm and 0.221 nm, and they are corresponded to (110) and (113) diffractions of Fe_2_O_3_, respectively. Lattices of β-FeOOH and Fe_2_O_3_ are both weak and hard to be observed by HRTEM (Fig. 3(a)), indicating their low crystallinity. This is why their diffraction peaks were not detected by XRD (Fig. 2(e)). When annealing temperature increases to 450 °C (Fig. 3(b)), compared with MA350, the diffraction spots are shaper and circles are more continuous, especially those for Fe_2_O_3_. Furthermore, the lattice fringes of Fe_2_O_3_ under HRTEM arrange more regularly, indicating that nanocrystallized Fe_2_O_3_ appears. When the annealing temperature further increases to 550 °C (Fig. 3(c)), diffraction spots in SAED pattern indicate that nanograins are almost monocrystallized Fe_2_O_3_, which are further confirmed by the HRTEM image in Fig. 3(c). It is known that β-FeOOH is an iron oxyhydroxide polymorph of ferric oxyhydroxide (FeOOH) with 4 double chains of FeO_3_(OH)_3_ octahedra [45]. When heated above 200 °C, β-FeOOH dehydrates and its lattices are disorganized, resulting in amorphous state after annealed at relatively low temperature (MA350); with the increased annealing temperature, new lattices assemble, forming Fe_2_O_3_ nucleus (MA450), and gradually grow into monocrystallized Fe_2_O_3_ (MA550) [43].

### 3.2 Physico-chemical properties and photothermal response of the coatings

Hydrophilicity and roughness of an implant surface play important roles in cell response and the subsequent tissue repair. Overall, compared with Ti control, coatings improve surface hydrophilcity, and different coating surfaces have no obviously statistic difference in contact angles (Fig. S3). All coating surfaces exhibit similar microporous topography with an average roughness of about 1 μm (Fig. S4).

**Fig. 4:**
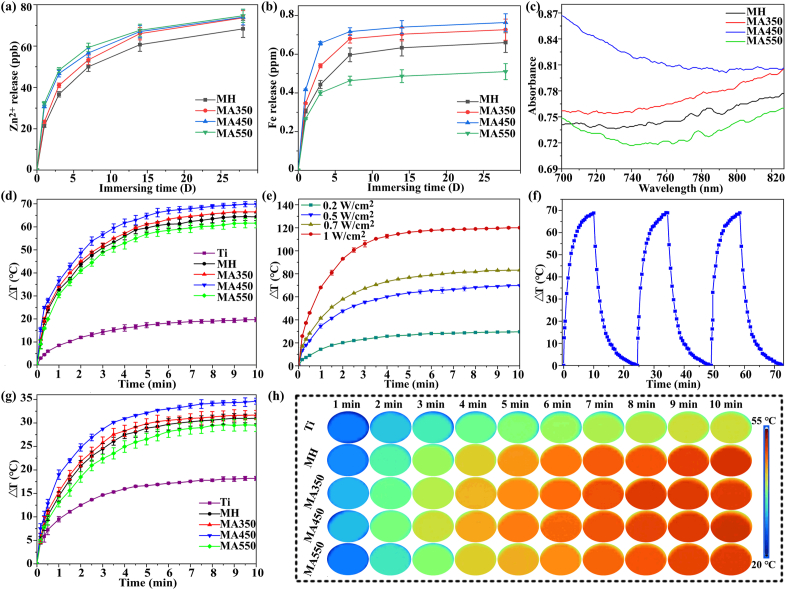
(a) Zn and (b) Fe concentrations released from different coatings after immersion for different times; photothermal property evaluation: (c) vis–NIR absorption spectra of different samples; (d) temperature changes of different coatings under 808 nm NIR irradiation in air with a power density of 0.5 W/cm^2^; (e) temperature changes of MA450 under 808 nm irradiation for 10 min with different power density; (f) temperature changes of MA450 under 808 nm NIR irradiation with 0.5 W/cm^2^ over 3 on-off cycles; (g) temperature changes of different coatings under NIR irradiation in 500 μl PBS, and (h) corresponding infrared thermal images of (g).

For cytocompatibility and antibacterial activity, Zn and Fe ions are both dose-dependent. They can improve fibroblast response to accelerate skin tissue repair in low concentrations, but kill bacteria and show cytotoxicity in high concentrations [2,5,42,46]. Therefore, the releasing of Zn and Fe ions from different coatings in physiological saline (PS) were measured (Fig. 4(a) and (b)). With the prolonged immersion time, accumulative Zn^2+^ and Fe^3+^ concentrations gradually increase, indicating their sustained release. At the initial immersion time (1, 3 and 7 d), Zn ions released from MA450 and MA550 are slightly more than those from MA350 and MH, but when the immersion is prolonged to 14 days, the accumulated Zn^2+^ ions released from different coating surfaces have no statistic differences. For Fe^3+^, at each immersion time, the accumulative release from different coatings almost followed the order: MA450 > MA350 > MH > MA550 (Fig. 4(b)). Nanosized β-FeOOH and Fe_2_O_3_ on MA450 have abundant of grain boundaries compared to the monocrystallized on MH and MA550, resulting in an easier release of Fe^3+^. So do MA350, which has amorphous β-FeOOH and Fe_2_O_3_ (Fig. 3(a)). β-FeOOH and Fe_2_O_3_ on MH and MA550 have similar size, and the variation in Fe^3+^ release should be due to different strength of intrinsically ionic bond between β-FeOOH and Fe_2_O_3_. Zn^2+^ shows antibacterial property through a mechanism of damaging bacterial membranes by generating reactive oxygen species [47]. Fe ion can inhibit bacteria via inducing Fenton reactions to damage the proteins and nuclear acids [48]. It is reported that minimum inhibitory concentration (MIC) of Zn^2+^ is 65 ppb and Fe^3+^ can inhibit the growth of *S. aureus* when its concentration is higher than 0.83 mM [41,49]. Zn^2+^ and Fe^3+^ release fastest from each coating after 1 d of immersion, but their concentrations are much less than the reported inhibition concentration against *S. aureus* and can't show antibacterial activity separately. However, Zn^2+^ in this concentration could improve adhesion, proliferation and phenotype of L929, which was confirmed in our previous work [5]. Similarly, tiny supplement of Fe^3+^ can promote proliferation and collagen deposition of fibroblast cell as well as integration with skin tissue [42]. So, it is deduced that Zn^2+^ and Fe^3+^ ions released from the coatings may improve fibroblast response and soft tissue integration.

Light adsorption of different coatings were recorded and shown in Fig. 4(c) and S5(a). Compared with MH, annealed coatings, especially MA450, show stronger absorption around the NIR region (Fig. 4(c)). Their bandgap energies were calculated and they are 2.13, 2.0, 1.65 and 1.70 eV for MH, MA350, MA450 and MA550, respectively (Fig. S5(b)). MA450 has the narrowest band gap of 1.65 eV, which corresponds to an absorption onset at 751 nm. It means that valence electrons in these coatings cannot be excited to conduction band by 808 nm NIR light, but the excited electrons can release energy in the form of heat. Fig. 4(d) and S6 show temperature changes of different coatings in air by NIR irradiation (808 nm, 0.5 W/cm^2^). With the prolonged irradiation, temperatures of all coatings increase rapidly, especially MA450. After 7 min of irradiation, temperature of each coating almost reaches a plateau. The temperature change at each irradiation time follows the order: MA450 > MA350 > MH > MA550 » Ti, which is consistent with their absorption strengthen at 808 nm. The increased temperature is 69.8 °C for MA450, whereas it is just 19.7 °C for Ti control after irradiation for 10 min. Photothermal properties are mainly depended on material phase [7,30,50], crystal structure [51] and grain size [52,53]. Apparently, nanocrystallized Fe_2_O_3_ and β-FeOOH increase NIR absorption ability and contribute the good photothermal performance of MA450.

Photothermal effect of a material doesn't only depend on the irradiation time but also relates to irradiation density. Fig. 4(e) and S7 show the temperature changes of MA450 with irradiation at 0.2, 0.5, 0.7 and 1 W/cm^2^ for 10 min, respectively. With the increased density, the temperature increases in various degree and it reaches 83.1 and 120 °C after irradiation at 0.7 and 1 W/cm^2^, respectively. Higher temperature should be beneficial to sterilize bacteria, but also impair cell function. So, irradiation density of 0.5 W/cm^2^ was selected for further study. Photo-stability is important for PTT. Fig. 4(f) shows photothermal response of MA450 under NIR irradiation for three on-off cycles as an example and Fig. S8 show representative real-time infrared thermal images during 20 min of irradiation. These indicate the good photo-stability of the coating. When implants service *in vivo*, NIR needs to pass through biological fluid and reaches implant surfaces. Fig. 4(g) shows photothermal performance of different samples in PBS. Temperature changes follow the similar order to those irradiated in air (Fig. 4(d)), but the plateau temperature for each surface is lower than those in air. For example, Ti hardly shows photothermal property and temperature increases by 18.2 °C after 10 min of irradiation, however, temperature of MA450 rises quickly, and it increases by 34.6 °C. Fig. 4(h) shows real-time infrared thermal images of different samples in PBS under continuous NIR irradiation for 10 min.

### 3.3 Antibacterial activity *in vitro*

**Fig. 5:**
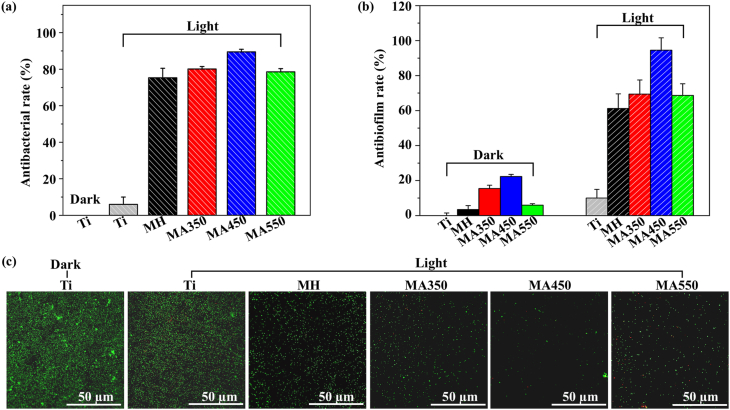
(a) Antibacterial ratios of different coatings after 808 nm NIR light irradiation; (b) *anti*-biofilm efficiencies of different coatings with or without NIR irradiation; (c) Live/dead staining images of biofilms on different samples.

It has been reported that highly local temperature (>50 °C) can induce bacterial proteins denature, resulting in irreversible bacterial destruction and death [[8], [9], [10]], but cells are injured if they experience excessively high temperature for long time. Local temperature and the duration time are very important to achieve good antibacterial activity and cell response. Based on the photothermal properties evaluated in PBS (Fig. 4(g)) and our preliminary experiment (Fig. S9), antibacterial activities of different samples were evaluated after NIR irradiation for 7 min, and Ti was used as a control (Fig. 5(a), S10). Ti shows no antibacterial efficacy against *S. aureus* even with irradiation. The irradiated coatings exhibit antibacterial properties in various degrees. MA450 can eliminate most of *S. aureus* (about 90%), and is orderly followed by MA350, MA550 and MH in accordant to the coating's photothermal response.

It is known that biological fluid containing abundant proteins, carbohydrates, and other substances, can facilitate adhered bacteria to form biofilm on implant surfaces. Once biofilms cover implant surfaces, it is very difficult for antibiotic to enter biofilms and not mention to eliminate them. Infections may persist and even induce implant failure. Consequently, the retardation and elimination abilities of biofilm on various surfaces were evaluated as shown in Fig. 5(b). Compared with Ti, without NIR irradiation, MH and MA550 haven't antibacterial ability, however, MA350 and MA450 can kill 15.5% and 22.3% of *S. aureus,* respectively. They are further confirmed by live/dead staining assay as shown in Fig. S11. Ti, MH and MA550 are almost covered with live bacteria colonies (stained green), however less live bacteria are observed on MA350 and MA450, and more dead bacteria (stained red) appear on MA450. As discussed in section 3.2, traces of Zn^2+^ and Fe^3+^ released from each coating can't kill bacteria separately (Fig. 4(a) and (b)), but a synergic effect of multi ions may show better antibacterial activity than separate ions in the same or even higher concentrations [5]. MA450 has the relatively more Zn and Fe release than other coatings after 1 d of immersion, resulting in its more activity in killing bacteria. The synergic effect of released ions in retarding biofilm formation was further confirmed by the antibacterial activity of extract liquids from different samples as shown in Fig. S12. After NIR irradiation, the elimination efficiencies of *S. aureus* biofilms by different samples show the similar order of their antibacterial activity in PBS: MA450 > MA350 ~ MA550 > MH » Ti, and MA450 has the highest elimination efficiency of 94.5% (Fig. 5(b), S13). The biofilms on different surfaces after NIR irradiation were also examined by live/dead staining assays (Fig. 5(c)). *S. aureus* on Ti with or without NIR irradiation were almost alive. On coating surfaces, green fluorescence decreased obviously, especially on MA450. The synergic effect of releasing ions and local high temperature endows MA450 with better biofilm elimination performance than other coatings.

### 3.4 Fibroblast response *in vitro*

**Fig. 6:**
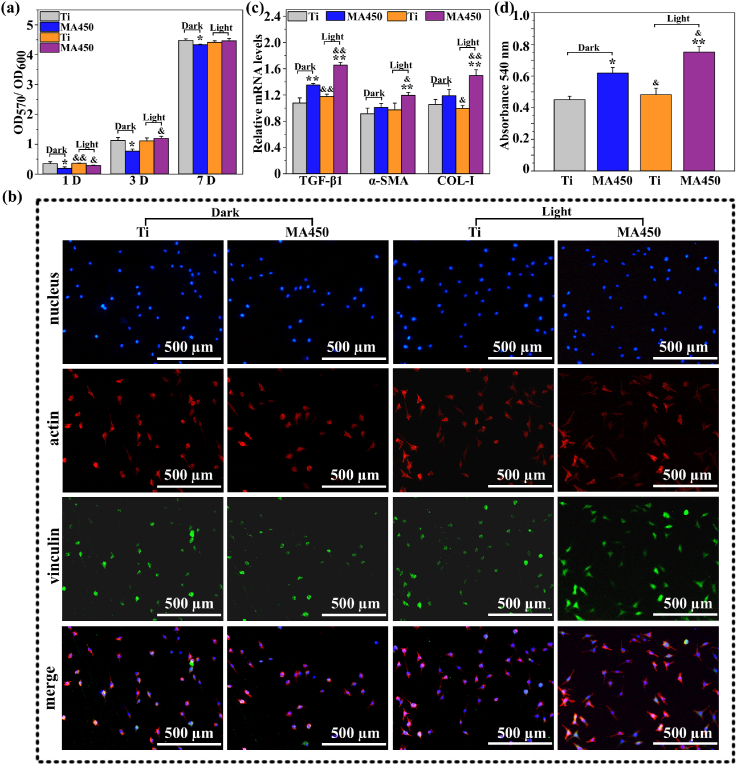
(a) Alamar Blue assays of cells cultured on different surfaces for 1, 3, and 7 days with or without NIR irradiation; (b) cell nucleus, actin and vinculin fiuorescence images of cells after 1 day of culture on different surfaces with or without NIR irradiation; (c) the gene expression of L-929 cultured on different surfaces after 7 days; (d) collagen secretion in the extracellular matrix after 7 days of incubation on different surfaces with or without NIR irradiation. *: p < 0.05 and **: p < 0.01 compared with Ti without NIR irradiation; &: p < 0.05 and &&: p < 0.01 compared with MA450 without NIR irradiation.

It is reported that mild heat stimulation (42 °C) can activate the heat shock promoter-mediated gene of MSCs, promote cell proliferation and differentiation, accelerate collagen deposition and improve tissue regeneration [13,14,54]. Based on the above antibacterial results (Fig. 4, Fig. 5), MA450 was selected to explore whether the coating accompanied with NIR irradiation could improve fibroblast behavior, and Ti was used as a control. The preliminary experiment (Fig. S14) indicates that the adhesion and proliferation of L-929 on MA450 could be enhanced after NIR irradiation for 30 s. So, irradiation time of 30 s was used to systematically study L-929 behaviors on MA450 with NIR irradiation (Fig. 6(a)). After incubation for 1 day, the mitochondria activity of cells on MA450 with NIR irradiation is higher than that without, but it has no significant difference compared to Ti with or without irradiation. After incubation for 3 days, mitochondria activity of cells on each surface all obviously increases, indicating cell proliferation. It is obviously higher on MA450 with NIR irradiation than that without. After incubation for 7 days, mitochondria activity of cells on all the coatings further increases and is almost the same on each surface. The morphologies of cells after incubation for 1 day were observed by nucleus-actin-vinculin staining as shown in Fig. 6(b). NIR irradiation doesn't change cell amount, actin and vinculin expressions on Ti obviously. MA450 has the least nuclei and vinculin expression, however, with NIR irradiation, cell amount, actin and vinculin expression all obviously increase. Furthermore, fibroblasts on MA450 with NIR irradiation almost display typically polygonal morphologies and organized filamentous actin bundles, indicating their good status. Overall, with NIR irradiation for 30 s, the increased temperature on Ti is feeble and can't influence fibroblast viability, but it is considerable for MA450 to improve fibroblast adhesion and proliferation.

The fibroblast function is very important for remolding soft tissue. Fig. 6(c) shows the expression of fibrogenic-related genes (TGF-β1, α-SMA and Col-I) to evaluate the phenotype of L-929 cells after incubation for 7 days. NIR irradiation doesn't work for cells on Ti, but MA450 promotes the expression of TGF-β1, α-SMA and Col-I obviously compared with Ti, especially with irradiation. Collagen secretion of fibroblast on different surfaces after 7 days of incubation was also examined (Fig. 6(d) and S15), and it follows similar order of expression of Col-I. These indicate that compared with Ti, a mildly increased temperature of MA450 by NIR irradiation is benefit for the functional expression of fibroblast and should promote bio-integration of Ti with surrounding tissue. In addition, trace of Zn^2+^ can promote the complex regulation of the sequence of signal molecules and mediators such as cytokines and growth factors, resulting the enhanced the adhesion and proliferation of fibroblasts [5]. Fe^3+^ is necessary for the assembly of Fe–S cluster proteins and acts as a cofactor in process of cell cycle, thus improving fibroblast response and accelerating skin tissue repair [46]. The released Zn^2+^ and Fe^3+^ contribute to the increased cell function on MA450 without irradiation compared with Ti control. The effect ratio of irradiation/released ions on cell function was estimated, using the formula, R=(L_B_-L_A_)/(L_A_-L_C_) × 100%, in which R is the ratio, L_B_ and L_A_ are the gene expression levels of MA450 with and without NIR irradiation respectively, L_C_ is the gene expression level of Ti without NIR irradiation, and the average R on gene expressions (Fig. 6(b)) is about 1.79. It indicates that the temperature increased by NIR irradiation plays a more important role in improving cell behavior, compared with the released ions.

### 3.5 *In vivo* experiments

**Fig. 7:**
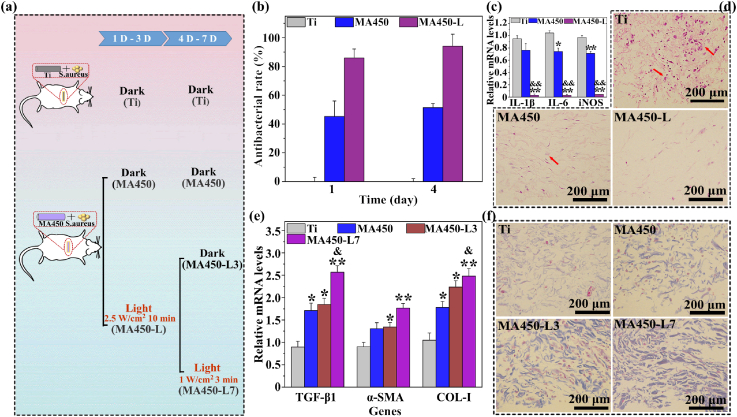
(a) Schematic illustration of the experiment *in vivo*; (b) antibacterial rates of the different samples with or without NIR irradiation after implantation for 1 and 4 days; (c) gene expression (IL-1β, IL-6 and iNOS) in surrounding skin tissue of different implants after 4 days of implantation; (d) H&E staining images for different samples after 4 days of implantation; (e) gene expressions (TGF-β1, α-SMA and COL-I) in surrounding skin tissue for different implants after 7 days of implantation; (f) Masson's trichrome staining images for different samples after 7 days of implantation. *: p < 0.05 and **: p < 0.01 compared with the Ti without NIR irradiation; &: p < 0.05 and &&: p < 0.01 compared with MA450 without NIR irradiation.

It is known that local environment surrounding implant *in vivo* is much more complicated than *in vitro*. NIR needs to go through soft tissue with a certain thickness and reaches the implant surface. So, the real intensity finally irradiating on implant surface should be lower that the initial setting [12,13,55]. Based on the local temperature requirements on bacterial sterilization and fibroblast response (Fig. 5, Fig. 6) as well as pre-experiments about attenuation of NIR light due to rat skin, the powers of NIR irradiation were set at 2.5 and 1 W/cm^2^ to achieve antibacterial and tissue regeneration performance respectively in infection models as schemed in Fig. 7(a). With a power of 2.5 W/cm^2^, local temperature of tissue with MA450 gradually rises with the irradiation prolonged, and finally reaches about 50 °C, as shown in Fig. S16, and the surrounding tissue can't be injured during the irradiation [9]. After 1 d of implantation, a multitude of bacterial colonies are observed on Ti (Fig. S17). MA450 without NIR irradiation can inhibit 40% of bacteria, which is due to the released Zn^2+^ and Fe^3+^ ions. With NIR irradiation, about 86% of biofilm is eliminated on MA450. When implantation is prolonged to 4 day, *S. aureus* colony on Ti decreases due to the natural immune response. With a synergic action of increased temperature, released ions and natural immune response, about 98% of biofilm is eliminated on MA450 with NIR irradiation (Fig. 7(b), S17). After 4 days of biofilm elimination by NIR irradiation, the biofilm didn't reform after 7 d of implantation (Fig. S18). Infection always induces inflammatory response, and the pro-inflammation-related genes (IL-1β, IL-6 and iNOS) from different tissues were detected after implantation for 4 days. Compared with Ti, MA450 down-regulates inflammatory response, especially with NIR irradiation, which is consistent with their antibacterial activity (Fig. 7(c)). Furthermore, H&E staining of tissue around samples were performed after 4 days of implantation (Fig. 7(d)). A large number of neutrophils and lymphocytes are observed for Ti control, whereas fewer inflammatory cells are found for the un-irradiated MA450 and hardly any inflammatory cells are observed for MA450 after irradiation. These results indicate that MA450 with NIR irradiation can eliminate the biofilm and reduce inflammation response significantly *in vivo*.

Based on the fibroblast behavior on different surfaces *in vitro* (Fig. 6), a part of MA450 implants were continuously NIR irradiated for another 4 days of implantation with a power of 1 W/cm^2^ for 3 min. With this power of irradiation, temperature of local tissue with MA450 *in vivo* gradually increased to about 42 °C, as shown Fig. S19. After implantation for 7 days, fibrogenic-related genes of TGF-β1, α-SMA and Col-I from tissue around the samples were analyzed (Fig. 7(e)). Due to the persistent infection, Ti shows low expression of TGF-β1, α-SMA and Col-I. Without NIR irradiation, MA450 with reduced biofilm and the released ions of Zn^2+^ and Fe^3+^, has increased gene expression levels, compared to Ti. With the initial elimination of biofilm, gene expression levels for MA450-L3 further increase, and even more for MA450-L7, which has a continued NIR irradiation for 4 days after biofilm elimination. Masson's trichrome staining was also carried out after 7 days of implantation. Deposited collagen surrounding the implants is stained in blue, and their amounts follow the same order of gene expression (Fig. 7(f)). It is reported that an increased local temperature (e.g. 42 °C) can increase bone repair in rat cranial defects, and promote the reconstruction of a full-thickness skin injury. We demonstrate that with a proper irradiation strategy, MA450 shows outstanding anti-infection performance and can rebuild tissue integration in an infection model efficiently. It is well explored that TiO_2_ by MAO can protect Ti implants from corrosion due to the tight adhesion of inner compact layer on Ti substrates [56,57]. HT generally can't change the interface of Ti and TiO_2_ obviously [15,41]. So, it is deduced that the composite coating on MA450 should not change the corrosion behavior of Ti substrate and it is a potential coating applied on percutaneous implants.

## 4 Conclusions

A multifunctional Fe_2_O_3_–FeOOH/TiO_2_ coating was prepared on Ti by MAO, HT and annealing treatment. The annealing treatment didn't change the surface morphologies and contents of Zn and Fe, whereas β-FeOOH gradually disorganized into amorphous state and transformed to Fe_2_O_3_ with the increased annealing temperature. Compared with monocryallized β-FeOOH (MH) and Fe_2_O_3_ (MA550) or their amorphorous state (MA350), nanocrystallized FeOOH and Fe_2_O_3_ gave MA450 relatively higher photothermal effect and more released ions. Even without NIR irradiation, MA450 showed weakly antibacterial activity and enhanced cell function, which were due to the released Fe^3+^ and Zn^2+^ ions. By regulating the NIR irradiation strategy, MA450 killed 90% of *S. aureus* in PBS, elminicate 94.5% of biofilm and accelerate fibroblast phenotype via up-regulating TGF-β1, α-SMA and Col-I expression *in vitro*. Simultaneously, after implantation for 4 days in an infected model, with NIR irradiation, 98% of biofilm on MA450 was eliminated and inflammatory response was reduced by down-regulating IL-1β, IL-6 and iNOS expression; after implantation for 7 days, TGF-β1, α-SMA and Col-I expression as well as collagen deposition were accelerated by the continued NIR irradiation, indicating an improved soft tissue integration on MA450. It is demonstrated that Fe_2_O_3_–FeOOH/TiO_2_ composite coating provides a new approach for timely eliminating biofilm and rebuilding soft tissue integration of percutaneous implant in an infected model.

## CRediT authorship contribution statement

**Yang Xue:** Software, Data curation, Validation, Writing – original draft, Methodology. **Jun Chen:** Validation. **Tiexin Ding:** Software. **Mengting Mao:** Visualization. **Shengbo Zhu:** Investigation. **Jianhong Zhou:** Methodology. **Lan Zhang:** Supervision, Conceptualization, Writing – review & editing. **Yong Han:** Writing – review & editing.

## Declaration of competing interest

The authors declare no competing financial interest.
