# Supplementary material for: Building biointegration of Fe2O3–FeOOH coated titanium implant by regulating NIR irradiation in an infected model
Source: Bioact Mater. 2021 Jun 29;8:1–11. doi: 10.1016/j.bioactmat.2021.06.029 (PMC8424078; doi:10.1016/j.bioactmat.2021.06.029)
Supplement: Multimedia component 1 [file mmc1.docx]

**Building biointegration of Fe_2_O_3_-FeOOH coated titanium implant by regulating NIR irradiation in an infected model**

Yang Xue^1^, Jun Chen^2,3^, Tiexin Ding^1^, Mengting Mao^1^, Shengbo Zhu^1^, Jianhong Zhou^4^, Lan Zhang^1^**^^[[1]](#footnote-1)^*^**, Yong Han^1*^

1. State-key Laboratory for Mechanical Behavior of Materials, Xi'an Jiaotong University, Xi'an 710049, China

2. Department of Osteology, Xi'an People’s Hospital ( Xi'an No. 4 Hospital), Xi'an, 710100, China.

3. Department of Osteology, Tangdu Hospital, Fourth Military Medical University, Xi'an, 710038, China.

4. Institute of Physics & Optoelectronics Technology, Advanced Titanium Alloys and

Functional Coatings Cooperative Innovation Center, Baoji University of Arts and

Sciences, Baoji, 721016, China

Table S1 The sequences of specific primer sets

| species | gene | forward primer sequence(5′−3′) | reverse primer sequence(5′−3′) |
| --- | --- | --- | --- |
| mice | α-SMA | CCTGAAGAGCATCCGACACT | AGAGTCCAGCACAATACCAGT |
|  | TGF-β1 | AGGAGACGGAATACAGGGCT | TTTGGGGCTGATCCCGTTG |
|  | Col-I | ACGCCATCAAGGTCTACTGC | CGTACTCGAACGGGAATCCA |
|  | GAPDH | CCACCCTGTTGCTGTAGCC | CCCACTCCTCCACCTTTGA |
| rat | IL-1β | GAACAACAAAAATGCCTCGTGC | GACAAACCGCTTTTCCATCTTCT |
|  | IL-6 | TGGAGTTCCGTTTCTACCTGG | GGTCTTGGTCCTTAGCCACTCC |
|  | iNOS | CACTGTGGCTGTGGTCACCTATC | ACTGACACTCCGCACAAAGCAG |
|  | α-SMA | ACCATCGGGAATGAACGCTT | CTGTCAGCAATGCCTGGGTA |
|  | TGF-β1 | GCTGAACCAAGGAGACGGAATA | GCAGGTGTTGAGCCCTTTCC |
|  | Col-I | CCCAGCGGTGGTTATGACTT | TCGATCCAGTACTCTCCGCT |
|  | GAPDH | CTGGAGAAACCTGCCAAGTATG | GGTGGAAGAATGGGAGTTGCT |


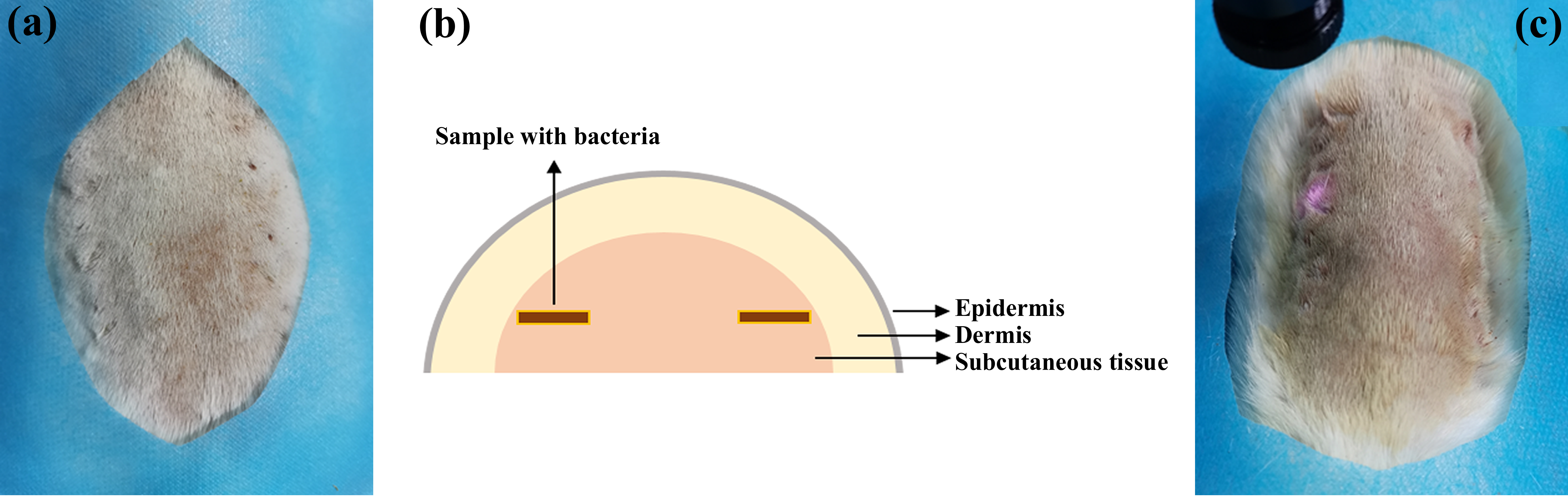


Fig. S1 Schematic diagram (a) and (b) showing the implanted region of samples in rat; (c) the process of 808 nm laser irradiation.

Table S2 Average Zn contents of different samples

| Samples | Zn contents (At %) |
| --- | --- |
| MH | 0.52 |
| MA350 | 0.49 |
| MA450 | 0.51 |
| MA550 | 0.50 |


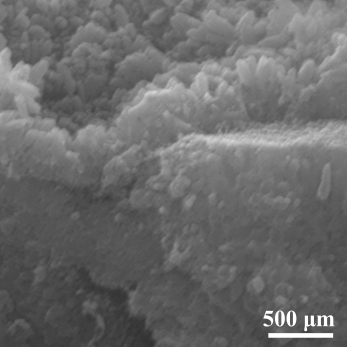


Fig. S2 The cross-sectional SEM morphology of MA450.

**The ratio of Fe_2_O_3_/FeOOH in MA450 was estimated as follows**.

Figure 2(g) shows the high-resolution spectra of O 1s for different surfaces. They were divided into three Gaussian-Lorentz component peaks. The peak at 529.8 eV is assigned to Fe-O; the peak at 531.2 eV corresponds to -OH in β-FeOOH; the peak at 532.1 eV attributes to the absorbed H_2_O. The relatively integral areas of the peak at 529.8 and 531.2 eV was used to estimate the relative ratio of Fe_2_O_3_/FeOOH by the formula, R=(A-C)/(B-D)×100%, in which R is the relative ratio of Fe_2_O_3_/FeOOH, A and C are the relatively integral areas of peaks at 529.8 eV for M450 and MH, B and D are the relatively integral areas of peaks at 532.1 eV for M450 and MA550, and the calculated ratio of Fe_2_O_3_/FeOOH is about 4.2.





Fig. S3 The contact angles and corresponding digital photographs of water droplets on different surfaces: (a) Ti, (b) MH, (c) MA350, (d) MA450 and (e) MA550. The wettability of different surfaces was measured by a surface contact-angle measurement machine (DSA30, Kruss, Germany). An image was captured and the contact angle was measured by the analysis software (DSAI). Three samples from each group were measured and two measurements were performed on each surface to obtain an average.





Fig. S4 The surface roughnesses of (a) MH, (b) MA350, (c) MA450 and (d) MA550. The surface roughness measurements of the samples were performed by a laser scanning confocal microscope (LSCM; VK-9710, Keyence, Japan).





Fig. S5 (a) UV–vis–NIR absorption spectra; (b) (hv)^2^ as a function of hv calculated based on the band gap energies of different coatings.





Fig. S6 Infrared thermal images of different samples exposed to 808 nm NIR irradiation with 0.5 W/cm^2^ for 10 min.





Fig. S7 Infrared thermal images of MA450 exposed to 808 nm NIR irradiation with different power density for 10 min.





Fig. S8 Infrared thermal images of MA450 exposed to 808 nm NIR laser on and off in period of 20 min.





Fig. S9 Antibacterial ratios of MA450 after 808 nm NIR light irradiation for different times.





Fig. S10 *S. aureus* colonies formed on different samples after 808 nm NIR light irradiation.





Fig. S11 Live/dead staining images of biofilms on different samples without NIR light irradiation.





Fig. S12 Antibacterial ratios of extract liquids from different samples.





Fig. S13 *S. aureus* colonies formed on Ti and different surfaces after 808 nm NIR light irradiation.





Fig. S14 Alamar Blue assays of L-929 cultured on MA450 with irradiation for different times after 1 and 3 days of incubation. MA450-0.33, MA450-0.5, MA450-1, MA450-2, and MA450-3 are MA450 samples with 808 nm NIR irradiation for 0.33, 0.5, 1, 2, and 3min, respectively.





Fig. S15 Staining pictures of collagen secretion in the extracellular matrix after 7 days of incubation on different coatings.





Fig. S16 Temperature change and infrared thermal images of MA450 exposed to 808 nm irradiation with 2.5 W/cm^2^ for different times.





Fig. S17 Bacteria colony images of different samples with or without irradiation after 1 and 4 days of implantation.


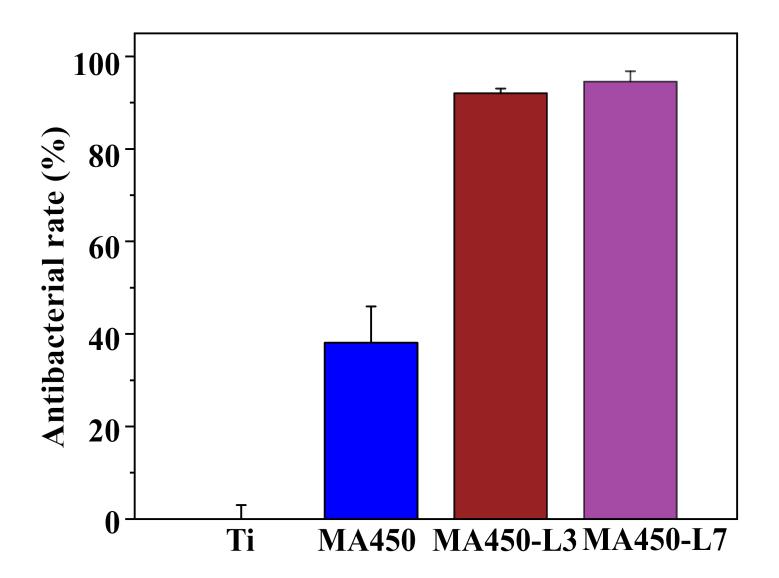


Fig. S18 Antibacterial rates of different samples with or without NIR irradiation after implantation for 7 days.





Fig. S19 Temperature change and infrared thermal images of MA450 exposed to 808 nm irradiation with 1 W/cm^2^ for 3 min in rat.

1. *Corresponding author, e-mail: lan.zhang@mail.xjtu.edu.cn (Lan Zhang), yonghan@mail.xjtu.edu.cn (Yong Han) Tel.:+86 02982665580; [↑](#footnote-ref-1)
